# Application of data fusion modeling for the prediction of auxin response elements in *Zea mays* for food security purposes

**DOI:** 10.5808/gi.22056

**Published:** 2022-12-30

**Authors:** Nesrine Sghaier, Rayda Ben Ayed, Ahmed Rebai

**Affiliations:** 1 Laboratory of Advanced Technology and Intelligent Systems, National Engineering School of Sousse, Sousse 4023, Tunisia; 2 Department of Agronomy and Plant Biotechnology, National Institute of Agronomy of Tunisia (INAT), 43 Avenue Charles Nicolle, 1082 El Mahrajène, University of Carthage-Tunis, Tunisia; 3 Laboratory of Extremophile Plants, Centre of Biotechnology of Borj-Cédria, B.P. 901, Hammam Lif 2050, TunisiaTunisia; 4 Laboratory of Molecular and Cellular Screening Processes, Sfax Biotechnology Center, B.P 1177, Sfax 3018,Tunisia

**Keywords:** AuxRE, data fusion method, prediction, *Zea mays*

## Abstract

Food security will be affected by climate change worldwide, particularly in the developing world, where the most important food products originate from plants. Plants are often exposed to environmental stresses that may affect their growth, development, yield, and food quality. Auxin is a hormone that plays a critical role in improving plants’ tolerance of environmental conditions. Auxin controls the expression of many stress-responsive genes in plants by interacting with specific cis-regulatory elements called auxin-responsive elements (AuxREs). In this work, we performed an *in silico* prediction of AuxREs in promoters of five auxin-responsive genes in *Zea mays*. We applied a data fusion approach based on the combined use of Dempster-Shafer evidence theory and fuzzy sets. Auxin has a direct impact on cell membrane proteins. The short-term auxin response may be represented by the regulation of transmembrane gene expression. The detection of an AuxRE in the promoter of prolyl oligopeptidase (POP) in *Z. mays* and the 3-fold overexpression of this gene under auxin treatment for 30 min indicated the role of POP in maize auxin response. POP is regulated by auxin to perform stress adaptation. In addition, the detection of two AuxRE TGTCTC motifs in the upstream sequence of the *bx1* gene suggests that bx1 can be regulated by auxin. Auxin may also be involved in the regulation of dehydration-responsive element-binding and some members of the protein kinase superfamily.

## Introduction

Maize (*Zea mays*) is a cereal plant and is one of the most widely distributed of the world's food crops, occupying an area of approximately 160 million hectares [1]. Maize is present in a variety of foods in the form of starch, proteins, lipids, vitamins, and minerals.

In recent years, climate change by devastating environmental changes has affected natural systems. In fact, environmental extremes and climate variability have enhanced the likelihood of plants experiencing numerous stresses. Plant physiology is strongly influenced by climate variability by several means.

Maize plants are often exposed to environmental stresses such as cold, drought, and high salinity that may affect their growth, development, yield, and food quality. To regulate these changes in their environment, plants respond by significant rearrangements in their transcriptomes and the modulation of the expression of numerous stress-related genes. Plant hormones have been reported to be involved in plants’ adaptation to different biotic and abiotic stress factors [2,3]. A plant hormone named auxin plays a critical role in improving plants’ tolerance to environmental conditions, both normal (e.g., water, nutrients, oxygen, and wind) and extreme (e.g., droughts, high salinity, high temperatures, and cold) [4-6].

Auxin has been found in all members of the plant kingdom [6,7], and it regulates many steps of plant growth such as cell division, cell elongation and cell differentiation [8], apical dominance [9], ethylene biosynthesis, root development [10], gravitropism, phototropism, and some other essential processes in plant development [11-14]. Auxin controls the expression of many stress-responsive genes in plants by interacting with specific cis-regulatory elements, called auxin-responsive elements (AuxREs), which are present in the promoter regions of these genes.

AuxREs, which contain the core TGTCTC motif, have been identified in the promoters of auxin response genes, and some of them have been confirmed *in vivo* [15,16]. The TGTCTC motif is also called the canonical AuxRE [17]. Many other variants of AuxREs have been reported to be auxin response factor (ARF) binding sites, such as TGTCCC, TGTCGG, and TGTCAC [11,18]. In fact, Boer et al. [19] indicated that the TGTCGG motif is more effective in binding ARF1 and ARF5. Additionally, a recent study of Galli et al. [20] indicated that the ARF clade A showed enrichment for the TGTCGG motif, whereas the ARF clade B showed enrichment for the TGTCCCC motif. Boer et al. [19] revealed that ARF DNA binding can involve either one or more binding sites. However, ARF binding is stronger and more frequent in sequences containing repeats of the TGTC motif [20]. AuxREs are key elements necessary in the auxin signaling network; therefore, identifying AuxREs is an important and significant step toward understanding the molecular basis of auxin’s actions.

In this work, we conducted an *in silico* prediction of AuxREs in the promoters of six auxin-responsive genes in *Z. mays*. We used microarray data on the auxin response in *Z. mays* to predict AuxREs in regulatory regions of these genes by applying a data fusion approach based on the combined use of Dempster-Shafer evidence theory and fuzzy sets to scan the upstream sequences of auxin response genes [21].

## Methods

### Microarray data analysis

To identify primary response genes regulated by auxin, we used microarray data analysis. We employed microarray data from the NCBI Gene Expression Omnibus (GEO) database (https://www.ncbi.nlm.nih.gov/geo/query/acc.cgi?acc=GSE15371). We selected genes whose expression was increased >3-fold at 30 min after treatment of *Z. mays* with 1 µM indole-3-acetic acid (IAA). The up- and down-regulated genes and their corresponding fold change values were obtained using Genevestigator software. The fold change values corresponded to the ratios of the hybridization signals of mock and IAA-treated plants [22]. A list of 62 early auxin-responsive genes displaying a more than 3-fold difference in fold change values was prepared. We analyzed the predicted AuxREs with the highest scores.

Upstream sequences of *Z. mays* genes were downloaded from the EnsEMBL database [23] using the RSAT-retrieve EnsEMBL sequence tool [24,25].

### AuxRE prediction methodology

In this study, we performed data fusion based on Dempster-Shafer theory and fuzzy set theory. We combined the predictive data extracted from two techniques that are frequently used to detect binding sites: linear discriminant analysis and overrepresented motif identification. Then, we applied our method to perform an *in silico* identification of AuxREs in the *Z. mays* genome.

Our approach involved two single hypotheses H1 (a motif is an AuxRE) and H2 (a motif is not an AuxRE), and one additional hypothesis H3, corresponding to the union of H1 and H2 and representing ignorance. The modeling process was performed through five major steps: (1) construction of two learning graphs, (2) determination of confidence regions based on the percentage of AuxREs that belonged to this region, (3) doubt modeling of the hypotheses, (4) fuzzification of the learning graphs, and (5) data fusion.

#### Construction of learning graphs

In the first step, a training set of validated cis-regulatory elements was collected from published articles and public databases. The available data were used as positive and negative training sets to build a discriminative model.

We extracted some parameters from each of the two predictive methods that were chosen to be combined by the Dempster-Shafer rule. We then created three learning graphs to elucidate the links between different parameters and types of motifs.

#### Determination of confidence regions

A graphical analysis showed that there was no clear discrimination of AuxREs from other types of motifs. Therefore, we chose to subdivide each graph into different regions, referred to as confidence regions, based on the percentage of AuxREs that belonged.

#### Modeling the doubt on the hypotheses

To give an automatic score to any unknown detected motif that would be located on the graph, we defined a gradual preference for each region through a set of four propositions: P4(Hi): total confidence in Hi; P3(Hi,Hj): strong preference for hypothesis Hi; P2(Hi,Hj): low preference for Hi; P1(Hi,Hj): total ignorance. The preference level for a hypothesis from P1 to P4 was then gradually represented by a mass value equal to 0, 0.33, 0.67, and 1, respectively [9,26].

#### Fuzzification of the learning graphs

The boundaries between regions were not very defined, and the transition from one region of the graph to another was smooth, not abrupt. Therefore, to have a continuous transition, we applied fuzzy logic theory by defining fuzzy sets for each measured feature to predict its membership degree for different possible parameters.

#### Data fusion methodology

For each detected motif, we attributed three mass values corresponding to the three learning graphs.


$$m\left(O\in S/P_{1}\&P_{2}\right)=\sum_{i}^{j}\mu_{P1\left(i\right)}\left(x\right)*\mu_{P2\left(j\right)}\left(y\right)*m_{R_{ij}}\left(O\in S/P_{1}\&P_{2}\right)$$

where S represents any subset of the hypotheses and $m_{R_{ij}}\left(O\in S/P_{1}\&P_{2}\right)$ designates the mass corresponding to the region Rij of two studied parameters on each graph. Next, we combined these masses by the orthogonal Dempster-Shafer sum.

## Results and Discussion

In our study, we focused on detecting AuxREs in the promoter regions of six early regulated genes in *Z. mays*. Detecting AuxREs in the upstream sequences of these genes may help to increase our understanding of the mode of action of auxin and provide guidance to elucidate the biological roles of some unknown genes in *Z. mays*. The studied genes were *GRMZM2G322819, GRMZM2G325693, GRMZM2G126772, GRMZM2G137341, GRMZM2G334165,* and *GRMZM2G085381*.

### Scan of the promoter of prolyl endopeptidase gene

The upstream sequence of prolyl endopeptidase protein (*LOC103646079/GRMZM2G322819*) contains a repeat of two AuxRE elements (TGTCTC) at positions –269 and –99. The prolyl oligopeptidase (POP) family is a group of serine peptidases capable of hydrolyzing peptides smaller than 30 residues. POP is present in most tissues and organisms, including humans and rats, and it plays interesting roles involving multiple biological processes such as signal transduction, protein secretion, and the maturation and degradation of peptide hormones. POP has been cloned from human lymphocytes [27], mouse brain [28], and porcine brain [29].

In plants, four members of the POP family of serine proteases have been identified: POP (EC3.4.21.26), acylaminoacyl peptidase (EC3.4.19.1), dipeptidyl peptidase IV (EC3.4.14.5), and oligopeptidase B (EC3.4.21.83) [30,31]. Their enzymatic properties have been characterized; however, the exact function of POP in plants is still unclear. Tan et al. (2013) [32] studied the expression of rice POP (OsPOP5) in *Escherichia coli* under different abiotic stresses. Expression of OsPOP5 enhanced the tolerance of *E. coli* to dehydration, heat, and high salinity, suggesting that OsPOP5 is a stress-related gene in rice and may play an important role in plant tolerance to abiotic stress [32].

Moreover, in *Coffea arabica*, prolyl oligopeptidase (CaPOP) is involved in the control of lateral shoot branching. In fact, differences in the expression of CaPOP in three cultivars of *C. arabica* L. are caused by one or several factor(s) that regulate their transcription. Auxins are known to influence axillary activity. Therefore, it is possible that CaPOP1 could interfere with the ability of auxin [33] to suppress axillary buds [34].

The detection of these AuxREs in the promoter of POP in *Z. mays* and the overexpression of this gene under auxin treatment (3-fold, 30 min) indicate the role of POP in the maize auxin response. POP is regulated by auxin to perform stress adaptation (Table 1).

### Scan of the promoter of the GRMZM2G325693 gene

The *LOC100277209* gene codes for an uncharacterized protein. It is a hypothetical protein predicted to be a transmembrane helix (https://www.uniprot.org/uniprot/A0A1D6HBE0). Auxin has a direct impact on cell membrane proteins. This short-term auxin response may be represented by the regulation of transmembrane gene expression. According to the research of Feng and Kim [35], after perceiving an auxin signal, ABP1 interacts directly or indirectly with some transmembrane protein. Auxin may also be involved in auxin transport.

The detection of a repeat of three canonical AuxREs at positions –964, –435, and –233 in the promoter of this gene confirms our suggestion (Table 1).

### Scan of the promoter of the benzoate carboxyl methyltransferase gene

The *LOC100282829* gene codes for a benzoate carboxyl methyltransferase (GRMZM2G126772) also named salicylate/benzoate carboxyl methyltransferase. This protein is a member of the plant methyltransferase family, which contains enzymes that work on a variety of substrates, including salicylic acid, jasmonic acid, and 7-methylxanthine. Moreover, it can catalyze the *N*-methylation of caffeine precursors [36].

Benzenoid carboxyl methyltransferases produce the methyl ester components of aromas in numerous plant species that are involved in plants’ communication with the environment [37].

Several plant hormones, such as auxins, cytokinins, abscisic acid, and gibberellins, include carboxyl-containing groups that can serve as methyl acceptors [38].

In *Arabidopsis thaliana*, methyltransferase is involved in the biosynthesis of methylsalicylate in response to stresses. It can use salicylic acid, benzoic acid, anthranilic acid, and m-hydroxybenzoic acid as substrates. The biological role of methyltransferase involves defense response, methylation, and response to wounding (https://www.uniprot.org/uniprot/Q6XMI3).

For several potential carboxyl substrates, it has been shown that the encoded protein preferably methylates the carboxyl group of the phytohormone IAA. Thus, some methyltransferase family members are implicated in chemically modifying auxin (IAA) [38].

In addition, some members of the carboxyl methyltransferase family (ATIAMT1) are involved in auxin homeostasis and IAA processing. In particular, this family is involved in converting IAA to its methyl ester form MelIAA (https://www.arabidopsis.org/servlets/TairObject?id=132987&type=locus).

In *Z. mays*, a repeat of four canonical AuxRE was detected in the upstream sequence of this gene (Table 1).

### Scan of the promoter of the dehydration-responsive element-binding gene

The *LOC100284491* (GRMZM2G137341) gene is a dehydration-responsive element-binding protein 1A. Furthermore, it is a putative AP2/ethylene responsive element binding protein (EREBP) transcription factor superfamily protein (https://www.uniprot.org/uniprot/A0A1D6HFV9).

An AP2 conserved domain was detected from 57 bp to 115 bp. AP2 is a DNA-binding domain that can be found in transcription regulators in plants such as EREBP. This domain binds to the 11 bp GCC box of the ethylene response element (ERE) promoter [39,40].

The expression of the DREB1/C-repeat binding factor (CBF) (A-1) group of DREB transcription factors is regulated at the transcriptional level. The expression of the majority of *DREB* genes is regulated by abiotic stresses, and the transcription of *DREB* genes is induced by different environmental factors. *DREB* genes are known to play crucial roles in responses to abiotic stress [41,42].

The DREB1/CBF family, has been shown to directly bind to the promoters of IAAs. Besides, *DREB2A* can directly regulate the expression of *IAA5* and *IAA19*, which are two desiccation stress-related genes [43].

Several plant hormones have been reported to be involved in stress signaling. Ethylene hormone serves as a key mediator of biotic and abiotic stress factors [44]. The conserved domain AP2/ERE superfamily plays an essential role in plant tolerance to biotic and abiotic stresses, such as cold and heat stress, ultraviolet light, drought, and salinity [45].

The promoter of this gene contains two predicted AuxREs (Table 1).

### Scan of the promoter of the protein kinase superfamily gene

The *LOC100279841* (GRMZM2G334165) gene codes for a putative protein kinase superfamily protein. A conserved domain located at 64 bp to 348 bp has been detected. This is a catalytic domain of the serine/threonine kinases, interleukin-1 receptor-associated kinases (IRAKs), and related serine/threonine protein kinases (STKs). STK proteins serve as an ATP binding site and are involved in the biological process of protein autophosphorylation (NCBI, UniproKb).

The IRAK subfamily is part of a larger superfamily that includes the catalytic domains of other protein STKs. STKs are involved in the regulation of auxin signaling. STK is induced by auxin. It plays the role of a positive regulator of cellular auxin efflux and controls organ growth by enhancing polar auxin transport. The protein kinase activity of PID is necessary for its role in the regulation auxin efflux carriers (https://www.uniprot.org/uniprot/O64682).

In addition, the *PINOID* gene, which is induced by auxin, encodes a protein-serine/threonine kinase. The protein kinase is found in vascular tissue in developing organs, as well as in leaves and floral parts [46]. Two TGTCTC motifs are present in the promoter of the putative protein kinase protein (Table 1).

### Scan of the promoter of the benzoxazinless 1 gene

This gene is benzoxazinless 1 (*bx1/ GRMZM2G085381*), which encodes a chloroplastic indole-3-glycerol phosphate lyase (a tryptophan synthase alpha chain trp1).

In maize, the TSA homolog BX1 catalyzes the synthesis of free indole from indole-3-glycerol phosphate, which is itself part of Trp-independent IAA production (https://www.uniprot.org/uniprot/P42390).

A study published by McMullen [47] claimed that the *bx1* to *bx5* genes are located on the short arm of chromosome 4S. However, the work of Frey et al. (1997) [48] assigned different bin positions from *bx1* to *bx5* on chromosome 4.

The hypothesis that variation at the *bx1* locus is responsible for DIMBOA production is less likely to be validated, and the biosynthesis of DIMBOA is controlled by nine genes including *bx1*, which represents the first one. Its role in DIMBOA biosynthesis is to govern the transcription of a key enzyme [49]. Polymorphisms within *bx1* were found to have the largest effect on DIMBOA content [50], causing the dominant allele to provide plants with substantial resistance against biotic stress [51].

A diversity analysis of 281 inbred lines of maize showed that *bx1* is likely to be responsible for much of the natural variation in the synthesis of DIMBOA (a benzoxazinoid compound) [50]. Maize resistance against many insect pests is influenced by genetic variation in benzoxazinoid content [52]. In addition, *bx1* is involved in the first step in the biosynthesis of benzoxazine, which improves resistance to pathogenic fungi, insect pests, and bacteria. Furthermore, *bx1*, which is a homolog of the alpha subunit of tryptophan synthase (TSA), is involved in tryptophan biosynthesis. *bx1* and TSA share a substrate, indole-3-glycerol phosphate, and a product, indole [53,54].

The detection of two AuxRE TGTCTC sequences in the upstream sequence of the *bx1* gene suggests that *bx1* can be regulated by auxin and is involved in the auxin response in *Z. mays* (Table 1).

### Summary

Food security will be affected by climate change worldwide, particularly in the developing world, thus affecting vulnerable people and their food systems. Stresses produced due to climate change and their impacts on crops needed to be managed through modern breeding technologies and biotechnological strategies to cope with climate change, in order to develop climate-resilient crops. Revolutions in genetic engineering techniques can also aid in overcoming food security issues exacerbated by extreme environmental conditions by producing transgenic plants.

Auxin is a plant hormone that plays a critical role in improving plant tolerance to environmental conditions, both normal (e.g., water, nutrients, oxygen, and wind) and extreme (e.g., droughts, high salinity, high temperatures, and cold) [4-6]. Auxin regulates many aspects of plant growth and development, such as cell division, cell elongation, and cell differentiation [8]; apical dominance [9]; gravitropism; phototropism; and some other essential processes [11-14]. In our study, we focused on the detection of AuxREs in the promoter regions of six early regulated genes in *Z. mays*. The detection of AuxREs in upstream sequences of these genes may improve our understanding of the mode of action of auxin and give guidance for further elucidating the biological roles of some unknown genes in *Z. mays*. The detection of an AuxRE in the promoter of POP in *Z. mays* and the 3-fold overexpression of this gene in response to auxin treatment for 30 min indicates the role of POP in the maize auxin response. POP is regulated by auxin to perform stress adaptation. In addition, the detection of two AuxRE TGTCTC motifs in the upstream sequence of the bx1 gene suggests that bx1 can be regulated by auxin. Furthermore, auxin is suggested to be involved in the regulation of dehydration-responsive element-binding, transmembrane protein expression, and some members of the protein kinase superfamily. This finding could serve as an innovative approach to solve the problem of maize adaptation in extreme environments and to ensure maize production in stress scenarios due to climate change, thereby achieving food security by using biotechnological tools such as molecular markers and bioinformatics modeling.

## Figures and Tables

**Table 1.** Predicted AuxREs in the promoters of the studied genes

Gene	Repeats	Start	End	matching_seq
Zm00001d002678	2	–274	–269	ccttTGTCTCctct
		–104	–99	tcccTGTCTCtagt
Zm00001d016948	3	–969	–964	aacaTGTCTCcgta
		–440	–435	tccgTGTCTCctca
		–238	–233	tcgaTGTCTCtaca
Zm00001d015217	4	–530	–525	ctcgTGTCTCgcgc
		–479	–474	ctcgTGTCTCgtgg
		–408	–403	ctcgTGTCTCgtgc
		–92	–87	acgcTGTCTCataa
Zm00001d017591	2	–521	–516	tcgcTGTCTCccgg
		–394	–389	cttgTGTCTCgtgc
Zm00001d020429	2	–528	–523	ctatTGTCTCcttg
		–368	–363	tccgTGTCTCttgg
Zm00001d048709	2	–941	–936	ttgtTGTCTCtggg
		–100	–95	cgctTGTCTCgaat

AuxRE, auxin-responsive element.
